# Non-contiguous finished genome sequence of *Staphylococcus capitis* CR01 (pulsetype NRCS-A)

**DOI:** 10.4056/sigs.5491045

**Published:** 2014-04-15

**Authors:** H. Lemriss, Martins Simões P, S. Lemriss, M. Butin, A. Ibrahimi, S. El Kabbaj, JP Rasigade, F. Laurent

**Affiliations:** 1 Department of Biosecurity PCL3, Laboratory of Research and Medical Analysis of the Fraternal of Gendarmerie Royale, Rabat, Morocco.; 2 International Centre for Research in Infectious diseases, INSERM U1111, University of Lyon, Lyon, France.; 3 Department of Clinical Microbiology, Northern Hospital Group, Hospices Civils de Lyon, Lyon, France.; 4 Medical Biotechnology lab. (MedBiotech), Medical and Pharmacy School, University Mohammed V Souissi, Rabat, Morocco.; 5 National Reference Center for Staphylococci, Hospices Civils de Lyon, Lyon, France.

**Keywords:** *Staphylococcus capitis* (NCRS-A), draft-genome, methicillin resistance, late-onset sepsis

## Abstract

*Staphylococcus capitis* is a coagulase-negative staphylococcus (CoNS) commonly found in the human microflora. Recently, a clonal population of *Staphylococcus capitis* (denominated NRCS-A) was found to be a major cause of late-onset sepsis (LOS) in several neonatal intensive care units in France. Here, we report the complete genome sequence and annotation of the prototype *Staphylococcus capitis* NCRS-A strain CR01. The 2,504,472 bp long genome (1 chromosome and no plasmids) exhibits a G+C content of 32.81%, and contains 2,468 protein-coding and 59 tRNA genes and 4 rRNA genes.

## Introduction

A frequent cause of low-weight newborns mortality and morbidity in Neonatal Intensive Care Units (NICUs) are late-onset sepsis (LOS), that are defined as sepsis occurring after 3 days of age. The most frequently encountered pathogens are coagulase-negative staphylococci (CoNS) and within those *Staphylococcus epidermidis* has been shown to be the most prevalent [1,2]. However, a few studies have reported the emergence of *Staphylococcus capitis* as a main CoNS- and LOS- causative pathogen in NICU settings [2-4]. A study in French NICUs [2] has demonstrated the spread of a single clonal population of methicillin-resistant *S. capitis* (pulsotype NRCS-A) associated to reduced susceptibility to vancomycin, the first line of antibiotics used in cases of LOS. Moreover, this clone has also been recently identified in NICUs in Belgium, United Kingdom and Australia, which suggests a worldwide distribution. In contrast, in adult bacteremia, *S. capitis* are rarely found and when detected, it presents a bigger diversity in terms of genotypes as well as antimicrobial susceptibility profiles than neonates bacteremia.

In order to elucidate the molecular mechanisms behind the wide spreading of the *S. capitis* NRCS-A clone in NICUs throughout the world, we sequenced a prototype strain (CR01).

### Classification and information

A strain belonging to the clonal population of *Staphylococcus capitis* NCRS-A pulsetype (Table 1) was isolated from the blood culture of a preterm infant with LOS, hospitalized in the NICU of the Northern Hospital Group Center (Hospices Civils de Lyon, Lyon, France) and suffering of LOS.

**Table 1:** Classification and general features of *Staphylococcus capitis* strain CR01, pulsetype-NRCS-A according the MIGS recommendation [5].

**MIGS ID**	**Property**	**Term**	**Evidence code**^a^
	Current classification	Domain *Bacteria* Phylum *Firmicutes* Class *Bacilli* Order *Bacillales* Family *Staphylococcaceae* Genus *Staphylococcus* Species *Staphylococcus capitis* Strain CR01, pulsetype-NRCS-A	TAS [6] TAS [7,8] TAS [9,10] TAS [11,12] TAS [13,14] TAS [11,15,16] TAS [17] TAS [2,18]
	Gram stain	Positive	TAS [19]
	Cell shape	Coccoid	TAS [17]
	Motility	Non-motile	TAS [19]
	Sporulation	Non-sporuating	TAS [19]
	Temperature range	Mesophilic	IDA
	Optimum temperature	37°C	TAS [17]
	Carbon source	Carbohydrates (glucose,sacharose,fructose,manitol,mannose)	TAS [17]
	Energy source	Chemoorganotropic	TAS [17]
	Terminal electron receptor	O_2_	TAS [17]
MIGS-6	Habitat	Skin of humans	TAS [17]
MIGS-6.3	Salinity	Physiological	TAS [17]
MIGS-22	Oxygen	Facultative anaerobes	TAS [17]
MIGS-15	Biotic relationship	Free-living	TAS [17]
MIGS-14	Pathogenicity	Opportunistic pathogen (Nosocomial bacteremia in premature neonates)	TAS [2]
MIGS-4	Geographic location	NICU Lyon, France	TAS [2]
MIGS-5	Sample collection time	2007	IDA
MIGS-4.1 MIGS-4.2	Latitude – Longitude	45° 45' 35" N 4° 50' 32" E	IDA
MIGS-4.3	Depth	Not applicable	IDA
MIGS-4.4	Altitude	162 m	IDA

a) Evidence codes - IDA: Inferred from Direct Assay; TAS: Traceable Author Statement (i.e., a direct report exists in the literature); NAS: Non-traceable Author Statement (i.e., not directly observed for the living, isolated sample, but based on a generally accepted property for the species, or anecdotal evidence). These evidence codes are from the Gene Ontology project [20].

Species identification of the bacterial isolates and antimicrobial susceptibility testing (AST) were performed, respectively, using Vitek MS (bioMérieux, Marcy l’Etoile), 16S rDNA sequencing, the automated BD Phoenix system (Becton Dickinson, Sparks, MD) and with Shimadzu-MALDI-TOF MS system (Shimadzu Corporation), as implemented on [21].

**Figure 1:**
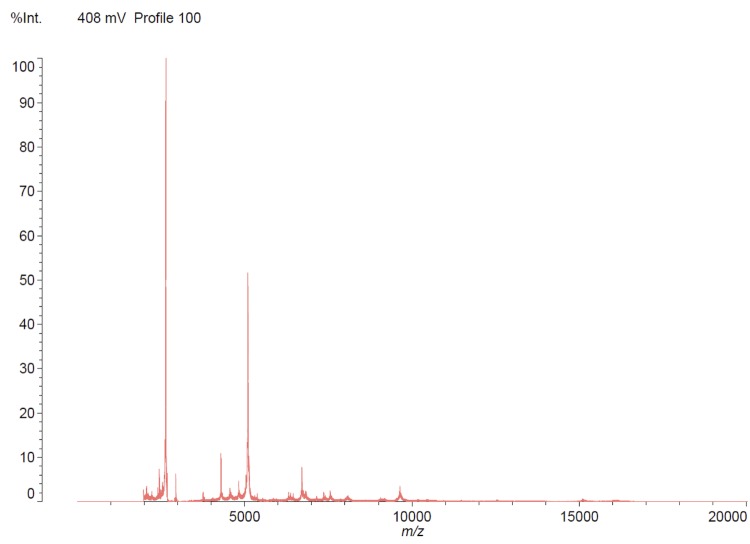
Reference mass spectrum from *Staphylococcus capitis* strain (CR01).

The strain was identified as being a Staphylococcus capitis* by* VITEK MS with 99.9% and at 93.7% by the MALDI-TOF MS, using the Shimadzu Launchpad software program and the SARAMIS database application (AnagnosTec GmbH) for automatic measurement and identification (Figure 1). Based on the information provided by the manufacture, when the score is ≥70%, identification is considered of high confidence.

The antimicrobial susceptibility test (AST) results were analyzed according to the recommendations of the French Microbiology Society [22]. The *S. capitis* bacteremia was considered positive based on a single positive blood culture [2,23]. The *S. capitis* NCRS-A isolate CR01, as all isolates from this clone, is resistant to penicillin, methicillin, gentamicin, rifampicin, hetero-resistant to vancomycin and sensitive to fusidic acid and fluoroquinolones.

**Figure 2:**
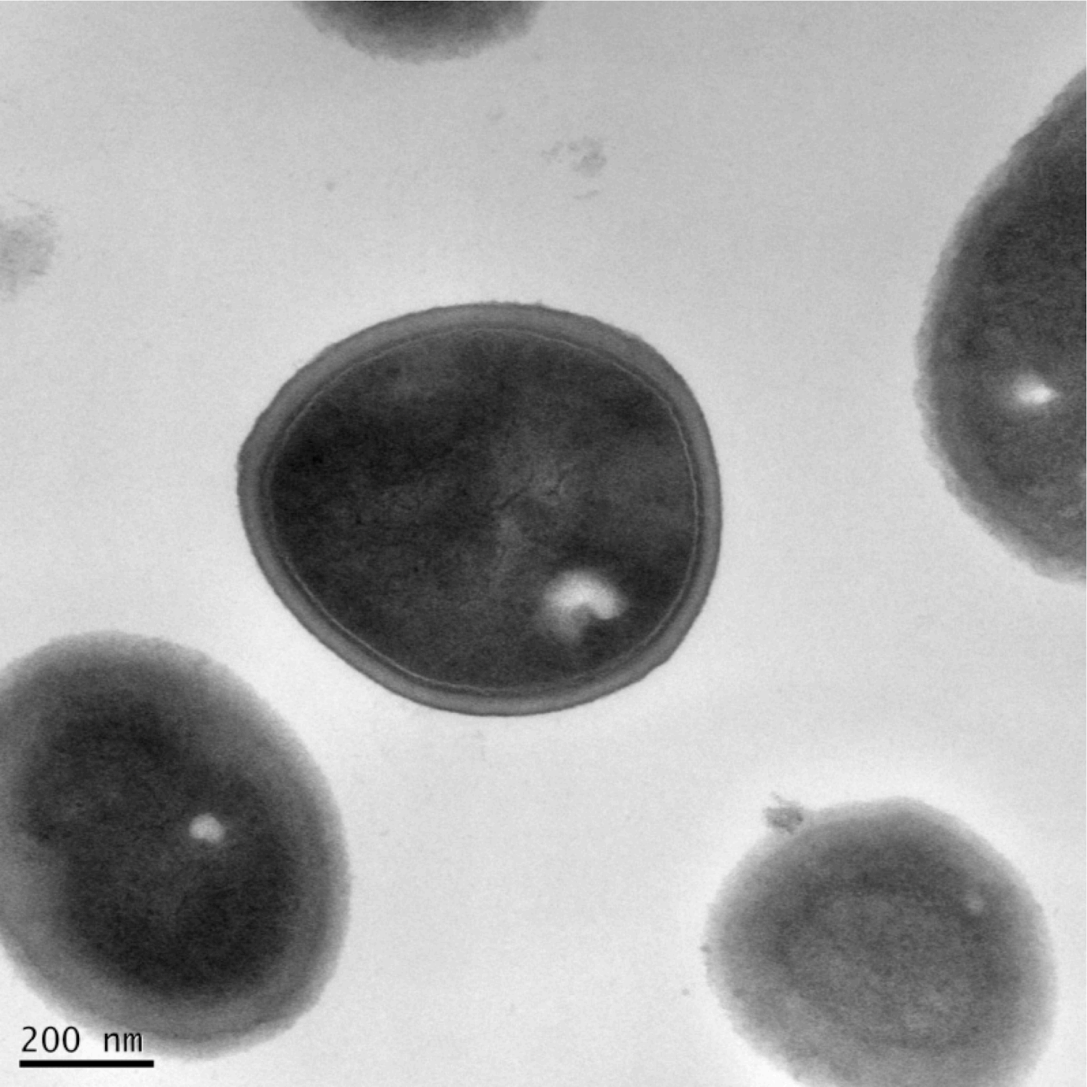
Transmission electron microscopy of Staphylococcus capitis strain (CR01) using a JEOL 1400. The scale bar represents 200 nm.

**Figure 3:**
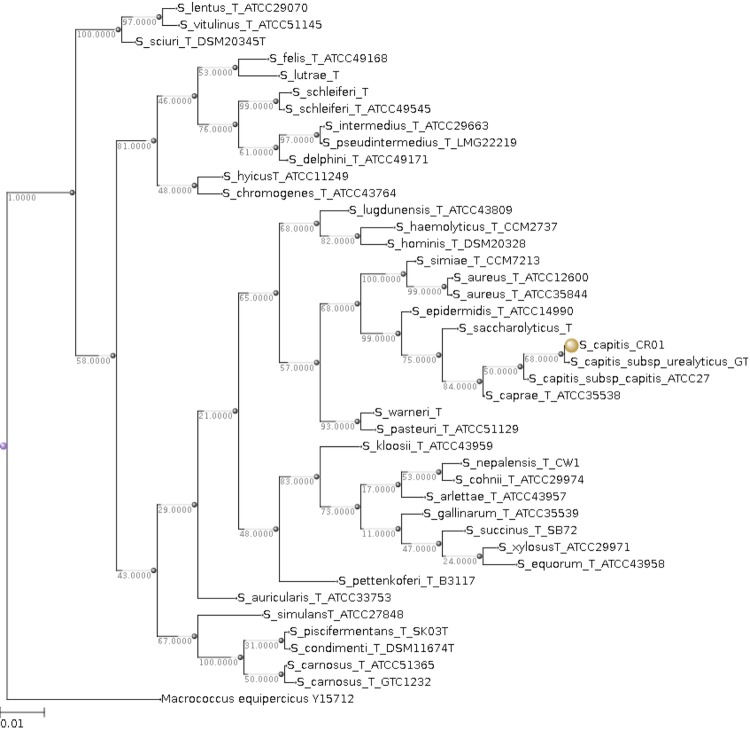
16S rRNA Phylogenetic tree highlighting the position of *Staphylococcus capitis* strain CR01 (indicated by the yellow circle) relative to other type strains within the genus *Staphylococcus*. All 16S rRNA sequences were obtained from the RDP database using as filtering criteria: sequences with more than 1200 nt and classified as “good” quality sequences. The tree uses sequences aligned with the MUSCLE software, with the default parameters as implemented on Seaview version 4 [24], and a tree was inferred based on 1285 sites using the distance model of observed divergence, as implemented in the BioNJ algorithm.

Table 1, Figure 2 and Figure 3 show detailed information concerning general features of *Staphylococcus capitis* strain (CR01) and position within the genus *Staphylococcus*.

The 16S rRNA sequences were aligned using the MUSCLE software, with the default parameters as implemented on Seaview version 4 [24], and a tree was inferred based on 1285 sites using the distance model of observed divergence, as implemented in the BioNJ algorithm, and a bootstrapping process repeated 500 times.

The final tree was rooted using the 16S rRNA sequence of *Macrococcus equipercicus* Type strain that belongs to a closely-related sister genus.

### Genome sequencing information

The genome sequence of *S. capitis* strain CR01 was determined by high-throughput sequencing performed on a Genome Sequencer FLX + system (454 Life Sciences/Roche) using FLX Titanium reagents according to the manufacturer's protocols and instructions, with approximately 47-fold coverage of the genome. This platform provides longer read lengths than other sequencing platforms to obtain raw sequences. *De novo* assemblies were performed using the Roche Newbler (v 2.7) software package.

### Genome project history

Table 2 presents the project information and its association with MIGS version 2.0 compliance [5].

**Table 2:** Project information

**MIGS ID**	**Property**	**Term**
MIGS-31	Finishing quality	Non-contiguous finished
MIGS-28	Libraries used	454 pyrosequence rapid library
MIGS-29	Sequencing platforms	454 GS FLX+
MIGS-31.2	Fold coverage	47.0 × pyrosequence
MIGS-30	Assemblers	Newbler Assembler 2.7
	GenBank	CBUB000000000.1

### Growth conditions and DNA isolation

The sample was prepared for sequencing by growing *S. capitis* CR01, aerobically at 37°C in Blood Agar for 24-48 hours. Genomic DNA was extracted using the PureLink^TM^ genomic DNA kit (Invitrogen^TM^) according to the manufacturer’s recommended protocol. The quantity of DNA obtained was determined using a NanoVue ^TM^ Plus (HVD Life Sciences), and 1 µg of DNA was used for sequencing of whole-genome of this strain.

### Genome sequencing and assembly

The isolated DNA of *S. capitis* CR01, was used to create 454-shotgun libraries following the GS Rapid library protocol (Roche 454, Roche). The resulting 454 DNA libraries were sequenced using a whole-genome shotgun strategy by GS FLX Titanium sequencing kit XL+ [25] (202,108 reads totaling 2.5 Mb, X48 fold coverage of the genome). Genome sequences were processed by Roche’s sequencing software according to the manufacturer's instructions (454 Life Science). The resulting shotgun reads were assembled *de novo* using the Roche Newbler assembly software 2.7 (454 Life Science) and 26 large contigs (Contig00001 to Contig00026) were obtained. The *N*50 was 176239 bp.

### Genome annotation

An automatic syntactic and functional annotation of the draft genome was performed using the MicroScope platform pipeline [26,27]. The syntactic analysis combines a set of programs including AMIGene [28], tRNAscan-SE [29], RNAmmer [30], Rfam scan [31] and Prodigal software [32] to predict genomic objects that are mainly CDSs and RNA genes. More than 20 bioinformatics methods are then used for functional and relational analyses: homology search in the generalist databank UniProt [33] and in more specialized databases as COG [34], InterPro [35], PRIAM profiles for enzymatic classification [36], prediction of protein localization using TMHMM [37], SignalP [38] and PsortB [39] tools.

## Genome properties

The genome includes one circular chromosome of 2,504,472 bp (32.81% GC content). A total of 2,565 genes were predicted with 2,453 being protein-coding genes, 59 tRNA-enconding genes, 4 rRNA-encoding genes (including 2 copies of 5S rRNA, 1 copy of both the large and the small-subunits, respectively, 23S and 16S rRNA) and 34 other RNA related ORFs. No plasmid was detected.

Of the 2,453 protein-coding genes, 1,892 genes (76.7%) were assigned to a putative function with the remaining annotated as hypothetical proteins. The predicted coding density in *S. capitis* strain CR01 *was 86%.*

**Figure 4:**
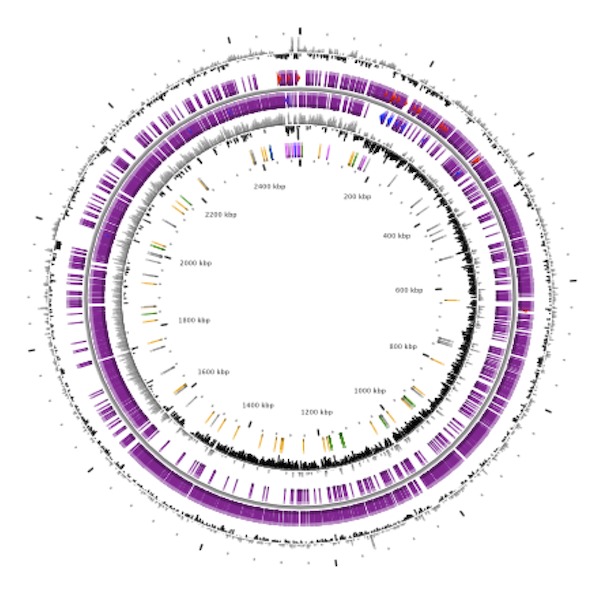
Graphical circular map of the chromosome. From outside to the center: Genes on the forward strand (colored by COG categories), genes on the reverse strand colored by COG categories), RNA genes (tRNAs green, rRNAs blue), GC content, and GC skew

Table 3 and 4 and Figure 4 detailed description of the properties and the statistics of *Staphylococcus capitis* strain CR01 genome. The distribution of the genes into COGs functional categories is presented in Table 5.

**Table 4:** Nucleotide content and gene count levels of the genome

**Attribute**	Value	% of total^a^
Genome size (bp)	2.504.472	100.00%
DNA G+C content (bp)	821.717	32.81%
DNA coding region (bp)	2.158.855	6.2%
Number of Scaffolds	26	-
Total genes^b^	2566	100.00%
RNA genes	97	4.00%
tRNA-enconding genes	59	2.30%
rRNA-encoding genes	4	0.20%
Protein-coding genes (CDS)	2454	96.00%
Genes assigned to COGs	1999	81.00%
Genes of unknown function	561	23.34%
Genes with transmembrane helices^c^	630	25.70%
CRISPR repeats	1	-

a) The total is based on either the size of the genome in base pairs or the total number of protein coding genes in the annotated genome.

b) Total number of genes includes CDS, RNA genes and pseudogenes.

c) Detection of transmembrane helices was performed using TMHMM v. 2.0 [40]

**Table 5:** Number of genes associated with general COG functional categories.

**Code**	**Value**	**%age**^a^	**Description**
J	178	7.21	Translation
K	184	7.46	Transcription
L	169	6.85	Replication, recombination and repair
D	29	1.18	Cell cycle control, mitosis and meiosis
V	85	3.44	Defense mechanisms
T	89	3.61	Signal transduction mechanisms
M	113	4.58	Cell wall/membrane biogenesis
N	23	0.9	Cell motility
W	1	0.04	Extracellular structures
U	33	1.34	Intracellular trafficking and secretion
O	86	3.48	Posttranslational modification, protein turnover, chaperones
C	144	5.83	Energy production and conversion
G	210	8.51	Carbohydrate transport and metabolism
E	370	14.99	Amino acid transport and metabolism
F	92	3.73	Nucleotide transport and metabolism
H	109	4.42	Coenzyme transport and metabolism
I	92	3.73	Lipid transport and metabolism
P	270	10.94	Inorganic ion transport and metabolism
Q	56	2.27	Secondary metabolites biosynthesis, transport and catabolism
R	425	17.22	General function prediction only
S	203	8.23	Function unknown
-	469	19.00	Not in COGs

**a)** The total is based on the total number of protein coding genes in the annotated genome.

## Conclusion

Here, we described a new genome sequence of *Staphylococcus capitis* (strain CR01 belonging to NRCS-A clone) as a first step toward comparing its content with other sequenced *Staphylococcus capitis* genomes as well as CoNS genomes of species associated with late-onset sepsis. Detailed analyses are in progress to identify virulence factors and mobile genetic elements (MBE), such as the staphylococcal chromosome cassette (SCCmec) [18], potentially related to the high specificity of the NRCS-A clone to the NICU environment.
